# Extracorporeal membrane oxygenation support in carinal resection for recurrent chondrosarcoma after previous distal tracheal resection

**DOI:** 10.1093/icvts/ivac148

**Published:** 2022-06-02

**Authors:** Luca Voltolini, Alberto Salvicchi, Giovanni Cianchi, Stefano Bongiolatti

**Affiliations:** Thoracic Surgery Unit, University Hospital Careggi, Florence, Italy; Thoracic Surgery Unit, University Hospital Careggi, Florence, Italy; Intensive Care Unit and Regional ECMO Referral Centre, University Hospital Careggi, Florence, Italy; Thoracic Surgery Unit, University Hospital Careggi, Florence, Italy

**Keywords:** Carinal resection, Extracorporeal membrane oxygenation, Tracheobronchial surgery

## Abstract

Carinal re-resection for tumour recurrence is rarely performed due to increased difficulty in airway reconstruction. We reported a successful case of carinal resection and reconstruction for recurrent chondrosarcoma after previous distal tracheal resection. Due to the technical complexity of the reconstruction and the poor respiratory reserve of the patient, the veno-venous extracorporeal membrane oxygenation support was used.

## INTRODUCTION

Carinal re-resection has been rarely performed and generally for complications (stenosis or anastomotic separation) of previous operation and very rarely for tumour recurrence [1], because of the increased difficulty in reconstructing the airway due to the length of resection. Here, we describe a case of carinal resection and reconstruction on veno-venous extracorporeal membrane oxygenation (v-v ECMO) after a previous distal tracheal resection, for a recurrent grade II chondrosarcoma.

## CASE REPORT

**Figure 1:**
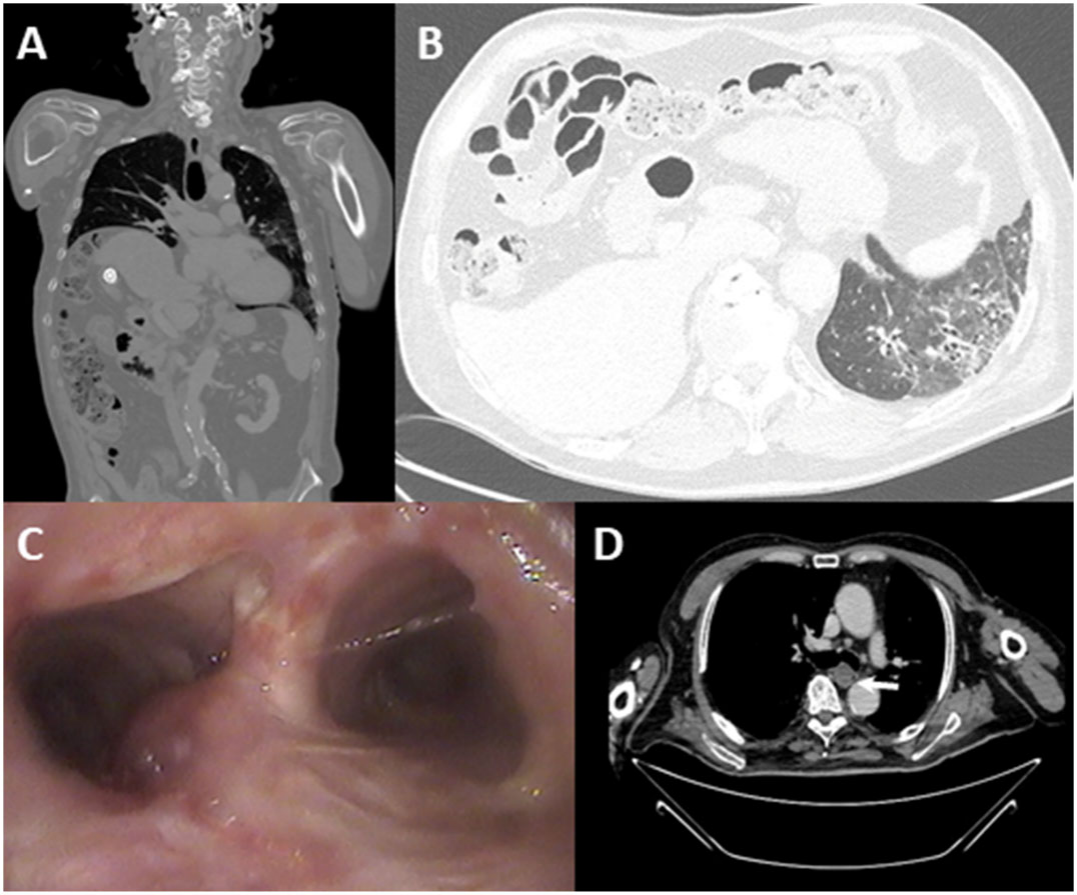
(**A**) Right hemidiaphragm elevation; (**B**) left lung fibrosis; (**C, D**) bronchoscopy and computed tomography images of chondrosarcoma.

A 78-year-old man presented with several-month history of cough and dyspnoea for minimal effort. He had undergone distal segmental tracheal resection (3 tracheal rings, about 1.2 cm) for a chondrosarcoma 10 years before at another hospital. Computed tomography scan demonstrated a 2.5 cm mass involving the carina and the proximal portion of the left main bronchus; an elevation of the right hemidiaphragm and a fibrotic left lung was also evident (Fig. 1). Bronchoscopy revealed an infiltrating mass in the distal trachea, extending to the origin of the left main bronchus. The patient was intubated with a single-lumen endotracheal tube and then placed on v-v ECMO (Getinge AB, Goteborg, Sweden); after a single dose of 5000 IU of Heparine, 19-Fr inflow cannula was directed into the right internal jugular vein and a 25 Fr outflow cannula into the right common femoral vein. Heparin was then infused, targeting an activated partial thrombostin time (aPTT) of 40–50, without reversing it. Then, a right posterolateral thoracotomy was performed in the fifth intercostal space, as at previous operation. Ventilation was stopped and the patient’s gas exchange was totally v-v ECMO dependent with a flow rate between 3 and 4 l/min without any problems of drainage or infusion, to facilitate the lysis of adhesions and mediastinal dissection.

**Figure 2:**
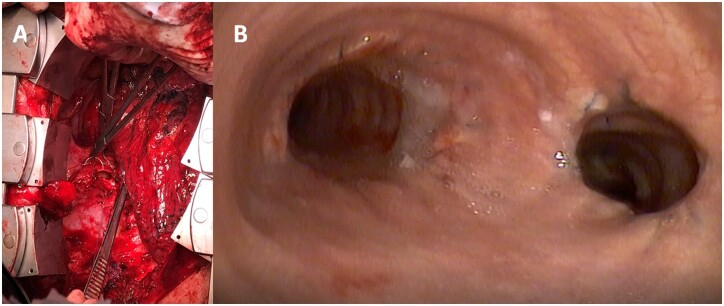
(**A**) Oval opening in the right lateral wall of the trachea; (**B**) bronchoscopy at 4 months after surgery.

After careful carinal dissection, the trachea and the right main bronchus were firstly transected under bronchoscopic guidance and lifted upwards to expose the left main bronchus distally to the lesion, where it was subsequently sectioned. The carinal specimen was then separated from any remaining mediastinal attachments with great care to preserve the left recurrent laryngeal nerve. The frozen section of tracheal and bronchial margins resulted negative for the presence of tumour. The pre-tracheal plane was bluntly dissected. After the intrapericardial release, 2 paired traction sutures on the tracheal and bronchial stumps were pulled-up together, in order to check the degree of tension. We opted for the Eschapasse technique [1], since the substantial amount of trachea resected during the 2 operations did not permit a neocarina construction approximating the median wall of the main bronchi. An end-to-end anastomosis between the distal trachea and the left main bronchus was performed by a 4/0 polypropylene single running suture, starting on the deepest site of tracheal and bronchial stumps. Then, an oval opening was made in the right lateral wall of the trachea, 2 tracheal rings above the prior anastomosis where the right main bronchus was implanted with the usual single running suture starting on the lower edge of the more anterior aspect of the anastomosis (Fig. 2A). The suture was left loose until the entire deepest aspect of the anastomosis was completed and then the reimplanted bronchus was pulled down onto the hosting structure, leaving the membraneous wall of the right main bronchus until last to allow balancing of any anastomotic calibre discrepancy and avoid as much as possible traction on it. The 2 anastomoses were covered with a ‘two tails’ pericardial flap after checking them for air leaks under saline as well as by bronchoscopy. The patient was extubated in the intensive care unit 3 h after surgery and the ECMO support was suspended.

The bronchoscopy on postoperative Day 7 showed no abnormal finding in the anastomotic sites. The postoperative course was uncomplicated and the patient was discharged after 8 days. At 6 months of follow-up, bronchoscopy showed intact and patent anastomoses (Fig. 2B) without recurrent disease. The final histology confirmed a grade II chondrosarcoma, with surgical margins free of disease.

## COMMENT

Carinal resection and reconstruction for recurrent chondrosarcoma after previous tracheal resection is exceedingly rare [2], also due to an increased risk of anastomotic dehiscence and separation [1]. The airway reconstruction should be carefully planned preoperatively; however, the optimal surgical technique can only be defined at surgical exploration. We adopted a right posterolateral approach that offers superb exposure and allows any type of reconstruction [1]. On surgical exploration, it was evident that the Eschapasse procedure could be the optimal reconstructive technique because the gap between the trachea and the left main bronchus was about 3 cm and the right main bronchus could be elevated to the lateral wall of the trachea, thanks to the elevation of the right hemidiaphragm and the intrapericardial release. ECMO was strongly indicated [3], since the fibrotic left lung did not allow a safe single lung ventilation. V-v ECMO was very effective, allowing prolonged apnoea for almost 3 h of surgery avoiding cross-field ventilation and making surgery easier with faster and more precise suture placement; on the other end, bleeding was not an issue with the low anticoagulation required by the v-v ECMO.

**Conflict of interest**: none declared.

## Data availability statement

The data underlying this article will be shared on reasonable request to the corresponding authors.
